# Sexual Partner Referral for HIV Testing Through Social Networking Platforms: Cross-sectional Study

**DOI:** 10.2196/32156

**Published:** 2022-04-05

**Authors:** Piao-Yi Chiou, Chien-Ching Hung, Chien-Yu Chen

**Affiliations:** 1 School of Nursing National Taiwan University College of Medicine Taipei Taiwan; 2 Department of Nursing National Taiwan University Hospital Taipei Taiwan; 3 Department of Internal Medicine National Taiwan University Hospital Taipei Taiwan; 4 Department of Tropical Medicine and Parasitology National Taiwan University College of Medicine Taipei Taiwan; 5 Department of Nursing National Taipei University of Nursing and Health Sciences Taipei Taiwan

**Keywords:** HIV testing, men who have sex with men, mobile health, motivational interviewing, referral and consultation, risk behavior, sexual partners, social networking

## Abstract

**Background:**

Men who have sex with men (MSM) who undergo voluntary HIV counseling and testing (VCT) often report condomless anal sexual intercourse, having many sexual partners, and being exposed to risky sexual networks. Limited research has discussed the application of motivational interviewing and convenience referral platforms to facilitate the referral of sexual partners for HIV testing among MSM.

**Objective:**

This study aimed to evaluate the effects of VCT referral by sexual partners through social networking platforms and the test results after elicited interviews with MSM; compare the characteristics and risk behaviors among MSM tested without referral, index subjects, and referred sexual partners; and explore unknown sexual affiliations through visualizing and quantifying the social network graph.

**Methods:**

This was a cross-sectional study. Purposeful sampling was used to recruit index subjects from a community HIV screening station frequented by MSM in Taipei City on Friday and Saturday nights. Respondent-driven sampling was used to recruit sexual partners. Partner-elicited interviews were conducted by trained staff before VCT to motivate MSM to become index subjects and refer sexual partners via the Line app, or to disclose the accounts and profiles of sexual partners on relevant social networking platforms. Referred sexual partners received rapid HIV testing, and the recruitment process was repeated until leads were exhausted.

**Results:**

After the interviews, 28.2% (75/266) of MSM were successfully persuaded to become index subjects in the first wave, referring 127 sexual partners via the Line app for rapid HIV testing and disclosing 40 sexual partners. The index subjects and tested sexual partners had more sexual partners (*F*_2_=3.83, *P*=.02), more frequent anal intercourse (*F*_2_=10.10, *P*<.001), and higher percentages of those who had not previously received HIV testing (*χ*^2^_1_=6.1, *P*=.047) compared with MSM tested without referrals. The new HIV-seropositivity rate among tested sexual partners was 2.4%, which was higher than the rate in the other 2 groups. The social network analysis revealed the following 4 types of sexual affiliation: chain, Y, star, and complicated. Among the HIV-negative sexual partners, 26.9% (43/160) had sexual affiliations with HIV-positive nodes, and 40% (10/25) were untested sexual partners with a direct sexual affiliation with an HIV-positive node. Four transmission bridges were found in the network graph.

**Conclusions:**

Partner-elicited interviews can effectively promote referral for HIV testing and case identification via Line, and can clarify unknown sexual affiliations of MSM to facilitate the development of a tailored prevention program. Social network analysis is needed for an insightful understanding of the different network structures.

## Introduction

### Background

Voluntary counseling and testing (VCT) for HIV, a process aimed at enabling an individual to make an informed choice regarding HIV testing, and condom use are key strategies for achieving the World Health Organization’s goal of 95% of all persons with HIV being aware of their status and being successfully treated by 2025, which is ultimately aimed at eliminating HIV by 2030 [1,2]. In 2019, it was estimated that 81% of people with HIV worldwide and 88% in Taiwan knew their infection status [3,4]. In addition to the current VCT model, a proactive approach should be applied to reach and test those who have not yet been diagnosed.

HIV could have been primarily transmitted through condomless sex in a social network characterized by similar and related risk behaviors [5,6]. Men who have sex with men (MSM) and who undergo VCT often report condomless anal sexual intercourse and having multiple sexual partners [7,8]. They comprise the main at-risk group for HIV transmission globally [9]. The active provision of VCT to the sexual partners of MSM who have been screened for HIV could promote the identification of HIV cases and the obstruction of transmission chains [10]. The referral of sexual partners is a delicate and private matter, and is difficult to compare against social network referral relating to nonsexual relationships [11,12]. There is a need for guiding frameworks for motivational interviewing on sensitive matters, including for sexual partners [13], and limited research has been conducted in this regard.

Motivational interviewing is based on the theory of helpfulness and disclosure, which emphasizes that self-disclosure and helping others could be conducive to interviewees being willing to share intimate topics like sexual relationships [14]. According to this theory, concepts, such as verbal persuasion, an emphasis on benefits, and the motivation to be a helper, contribute to the generation of interview content that encourages MSM to refer their sexual partners for VCT [15,16]. A convenient platform for initiating referrals is also necessary [17].

Social networking platforms that include mobile instant messaging apps (eg, Line and WeChat), mobile geosocial network apps (eg, Grindr, Jack’d, and Hornet), and web-based communities (eg, Facebook, Twitter, and Instagram), have become popular platforms for MSM to seek and meet sexual partners and receive health information [18-21]. Previous studies confirmed that posting screening advertisements, sending messages, or engaging in web-based discussions can effectively improve self-reported HIV testing behaviors among people engaging in condomless sex [22-25]. Such platforms provide a convenient private approach for delivering testing messages, and as the testing process is coordinated directly among MSM, their sexual partners, and testing staff, they could be used as referral platforms. Cross-referral between MSM and their sexual partners via social networking platforms presents unique opportunities to access connected communities and intervene in their risk-taking behavior.

Social network analysis is usually applied to reveal previously unknown facets about risky behaviors, specifically regarding affiliations between an individual (node) and the rest of the network [5,6]. The analysis of each node, which is defined as an individual engaging in certain types of actions in a network, provides valuable information on individual risk features and behaviors in a social network [26]. A social network graph can be developed for more diversified analyses, such as presenting the network type, and quantifying links between HIV-negative and HIV-positive nodes to understand the possible distances of HIV transmission [27].

### Objective

The purpose of this study was to evaluate the effects of sexual partners’ referrals for HIV testing through social networking platforms, along with the generated test results, after conducting motivational interviewing with MSM. Specifically, we compared differences in the characteristics and risk behaviors of the MSM tested without referral, index subjects, and referred sexual partners in this network. Finally, we aimed to reveal the unknown sexual affiliations of each HIV-negative node to HIV-positive nodes through visualizing and quantifying the social network graph.

## Methods

### Study Design

The study followed a cross-sectional design. Purposeful sampling was used to recruit participants, while respondent-driven sampling was used to recruit sexual partners, who were referred via social networking platforms. Partner-elicited interviews were conducted until no new sexual partners were referred or no social networking accounts and profiles of sexual partners on platforms where they first met were disclosed by the index subjects to the research team.

### Participants

Participants included those who had undergone VCT by trained staff, had self-identified as MSM, had engaged in condomless anal sexual intercourse, were over 20 years old, were literate, owned a mobile phone, lived in Taipei, and had agreed to participate in this research. Men self-identifying as transgender were excluded. Participants who received VCT during the first wave and referred or disclosed their sexual partners via social networking platforms were defined as index subjects, while sexual partners were defined as those with whom the referrer had engaged in sexual intercourse during the previous 3 months. Tested sexual partners were defined as those who had been referred through the referrer and who had completed the rapid HIV test by the trained staff. This process continued until all leads were exhausted. Participants who were unwilling to refer or disclose their sexual partners during the first wave were defined as MSM tested without referrals.

### Procedure and Data Collection

This research was conducted from August 2017 to January 2018. MSM who underwent VCT and met the inclusion criteria were recruited at a community screening station in a gay village, mainly a business community, frequented by MSM in Taipei City on Friday and Saturday nights from 6 PM to 10 PM. The reason for choosing this location is that the station provides HIV screening services for the target group from New Taipei City and Taipei City, which have the highest HIV prevalence rates in Taiwan. After explaining the research purpose and procedure, written informed consent was obtained from the participants. Individuals who agreed to participate in the research then completed a questionnaire, and a pretest counseling session was conducted by trained staff. The 20-30–min partner-elicited interviews were performed and information on the sexual partners was collected by the same staff. A free and anonymous rapid HIV test and posttest counseling session were subsequently provided to the participants. The reasons for refusal to be recruited, or not agreeing to refer or disclose any information on sexual partners, were also discussed and recorded in the first wave. For those who refused to participate in the research, the HIV testing procedure was completed without the partner-elicited interview.

**Figure 1:**
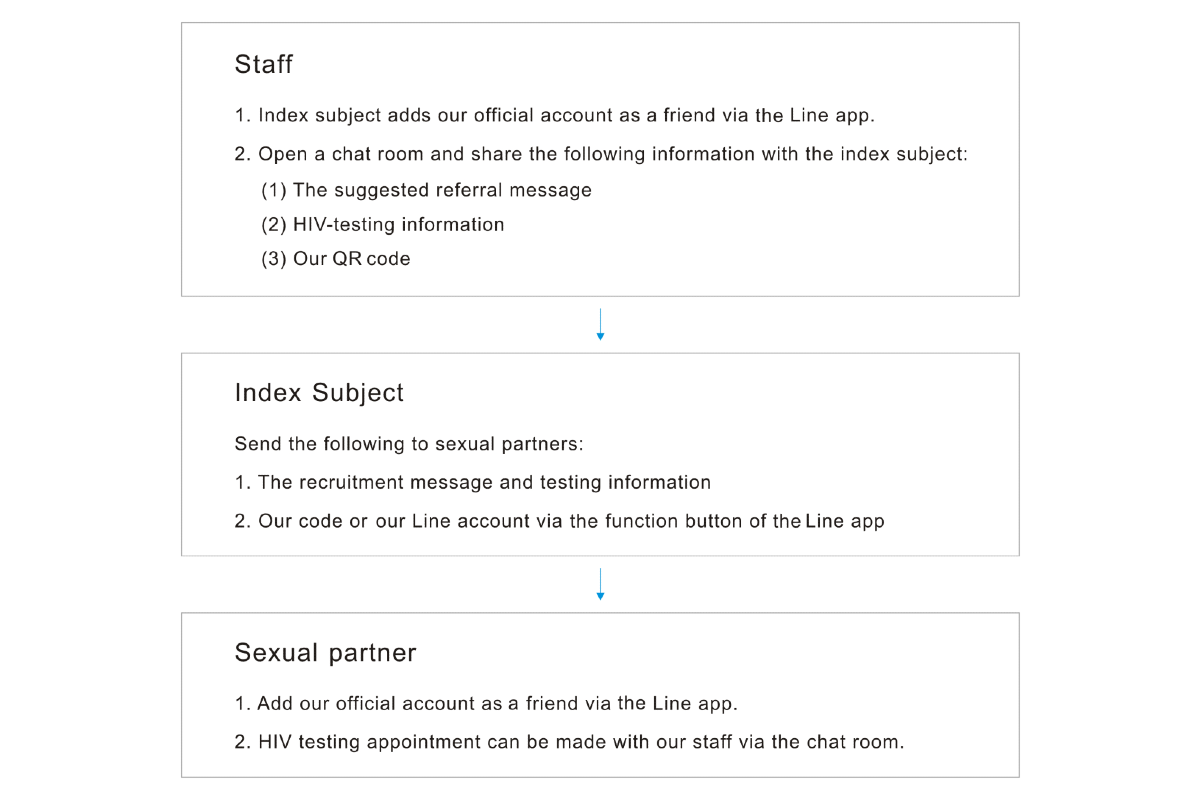
Flowchart of the referral process using the Line app.

**Figure 2:**
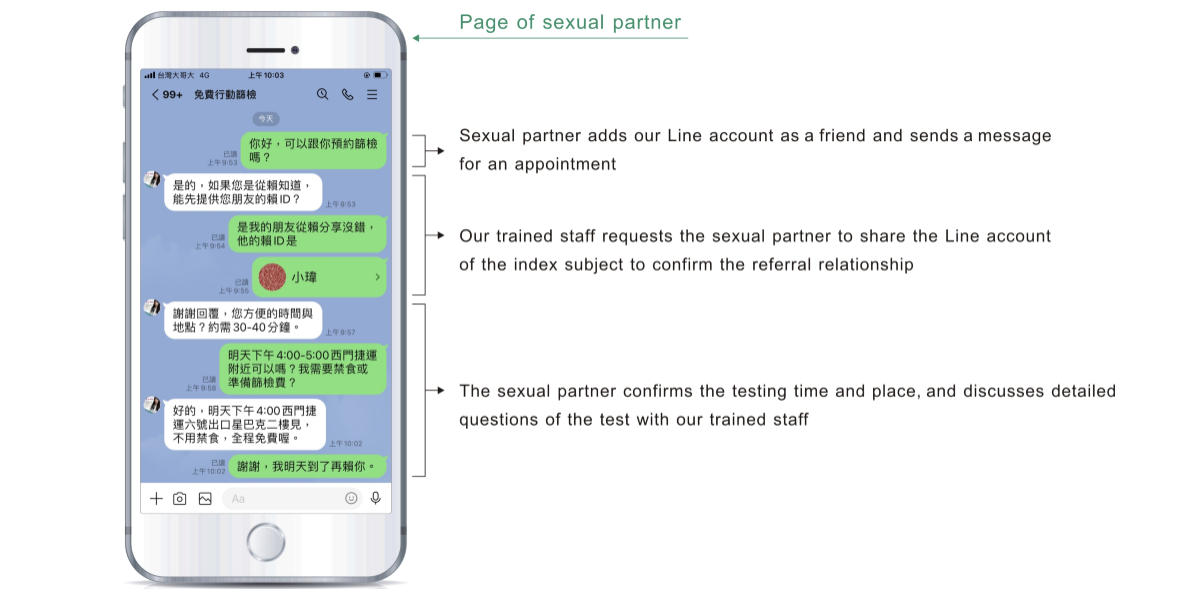
A screenshot of confirmation of referral and the testing appointment.

The Line app is one of the most popular mobile instant messaging apps in Taiwan. It can be downloaded free of charge and was therefore used as a referral platform in this study. Successful referrers were added as friends to our official Line account, to open a chat room and send messages. Then, a QR code for our official Line account was provided to index subjects. They could post a message to share the QR code or our official Line account directly to their sexual partners via the in-app function. After reading our recruitment message, the sexual partners could then add our official account as a friend and make an appointment for HIV testing with our staff through the chat room. The referral process is depicted in Figures 1 and 2.

Partner-elicited interviews were conducted with the tested sexual partners to encourage further referrals. Sexual partners’ test appointments and results were checked weekly. If the referred sexual partner did not contact our official Line account, the referrer was requested to remind the partner to contact us.

The trained staff accompanied and referred all participants with HIV-positive results to the HIV case manager at the hospital for HIV confirmation, diagnosis, assessment, and treatment.

#### Partner-Elicited Interviews

The partner-elicited interviews were designed around the theoretical concept of helpfulness and disclosure [14-16] (Multimedia Appendix 1). The interview process proceeded smoothly as it commenced with an empathic and inspiring statement, followed by an in-depth discussion guided by open-ended questions [28]. Participants were motivated to become referrers via verbal persuasion, which promoted self-awareness about their HIV status and facilitated discussions on safe sexual behaviors and HIV prevention measures in partner relationships. The benefits for their referred sexual partners were then explained, including 2 easy steps via the Line app to link their sexual partners with our staff to make an appointment and receive a free and anonymous rapid HIV test in a convenient and personalized manner. If the participants were unwilling to directly refer their sexual partners, the staff elicited them as helpers by asking them to disclose information about their sexual partners, such as their accounts and profiles on specific social networking platforms, and their current HIV status, thus helping to discover unknown HIV-related sexual affiliations within the MSM community.

The content validity of partner-elicited interviews was examined through theoretical consistency and expert validity. The trained staff who conducted the interviews had completed a 5-hour training course related to HIV testing, counseling, and practice, presented by the Taiwan AIDS Nurse Association, and an additional 8 hours of internship for consistency training before participating in the formal research.

#### The Demographics, Risk Behaviors, and HIV-Testing Experience Questionnaire

The demographics portion of the questionnaire asked participants about their age, education, employment, religion, marital status, sexual orientation, and sexual preferences, while the risk behavior section asked about their experiences with finding sexual partners through social media, age of sexual debut, number of sexual partners in the past 3 months, frequency of anal intercourse and condom use in the past 3 months, and experiences with recreational drug use. The HIV-testing experience questionnaire asked about experiences with HIV testing and frequency of HIV testing.

#### Sexual Partner Record

A data collection form was used to record information about referred/disclosed sexual partners of index subjects. Contents included nicknames and/or contact information (eg, user account and profile information on social media platforms or the last 4 digits of their phone number), or any other information about sexual partners, including the selection of HIV testing appointments (descriptions of the time/location/date and any special requirements for delivering an HIV test) and the current HIV status.

#### Rapid HIV Testing Equipment

The third generation of the Alere Determine HIV-1/2 was used to test for HIV-1/HIV-2 antibodies in human serum or plasma. It was licensed by the Taiwan Food and Drug Administration, with a sensitivity of 99.75% and a specificity of 99.87% [29]. Tests took approximately 15 minutes to complete, and the test results were managed and recorded by trained staff.

### Analysis Procedure

The primary outcome was HIV-positive results. Secondary outcomes were the referral rate; the comparison of demographics, risk behaviors, and HIV-testing experiences; and quantified findings of the social network graph. Demographic data were analyzed by calculating the means of continuous variables and the percentages of categorical variables. A one-way analysis of variance, Fisher least significant difference test, and chi-square test were used to investigate the differences and associations in participants’ characteristics, risk behaviors, HIV-testing experiences, and HIV test results between different groups.

The UCINET software package [30] was used to analyze the social network at the ego level and the output of the social network graph. A node was defined as an individual in the network. The numbers of the intermediate nodes linking each HIV-negative node of tested and untested sexual partners to the closest HIV-positive node were calculated (the calculation was not repeated), to present the influence of each node in the possible distance of HIV transmission among the social networking platform users. Unique codes were used within the network graph to maintain anonymity.

### Ethical Statement

This study was approved by the institutional review board of MacKay Memorial Hospital (17MMHISO68e). Written informed consent was obtained from all participants, who were given information about the study’s purpose, procedures, potential benefits and harms, and confidentiality. All information was collected and processed anonymously and replaced with unique codes. At the end of the research, all the accounts and records of the research participants and untested sexual partners were deleted to protect their privacy. Each participant received a compensatory voucher worth US $6.66 after completing their interviews and rapid HIV tests. Participants who successfully referred their sexual partners received an additional voucher of the same value.

## Results

During the study period, 279 MSM visited our screening stations for rapid HIV tests, all of whom met the inclusion criteria. Of these, 4.7% (13/279) refused to participate owing to not having enough time to attend the interviews and 95.3% (266/279) agreed to participate in the partner-elicited interviews, with 28.2% (75/266) agreeing to refer/disclose information about their sexual partners and become index subjects. The remaining 71.8% (191/266) of subjects without referral provided the following reasons for declining: 32.5% (62/191) reported that their sexual partners had recently undergone HIV tests, 30.4% (58/191) preferred to respect the privacy of their sexual partners, 25.7% (49/191) did not have any information about their sexual partners, and 11.5% (22/191) simply declined to answer.

**Figure 3:**
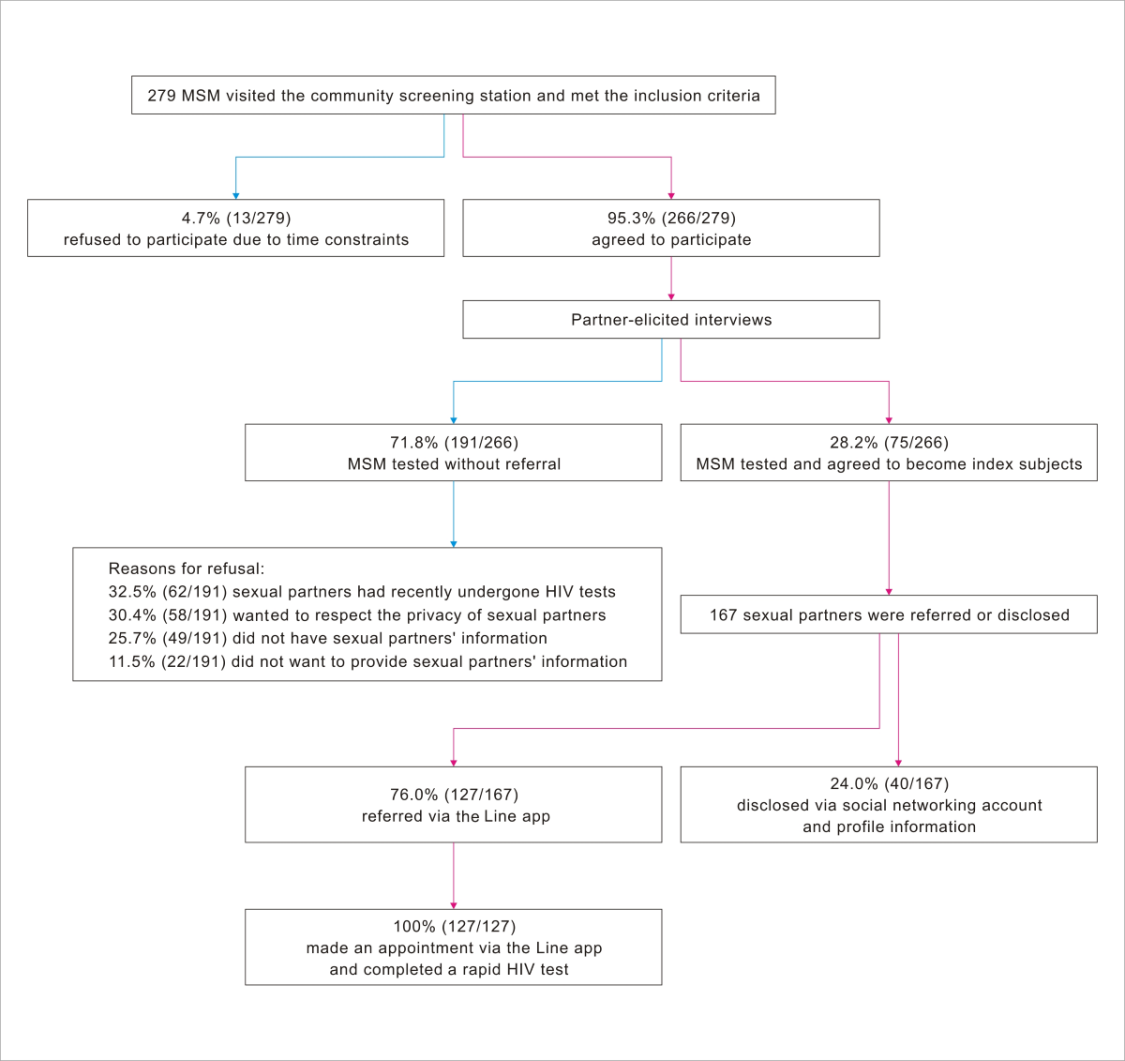
Diagram of the recruitment process. MSM: men who have sex with men.

At the end of the recruitment period, a total of 167 sexual partners were referred/disclosed, and of these, 127 had been referred via the Line app and 40 were disclosed by index subjects. All 127 who were referred via the app made appointments and completed the rapid HIV test conducted by our trained staff, at a time and place designated for their convenience. As for the 40 partners who had not been referred for HIV testing, their social networking accounts and profiles on platforms where the index subjects first met them were disclosed by the index subjects, including geosocial network apps (Hornet: 14/40, 35.0%; Grindr: 7/40, 17.5%; and Jack’d: 4/40, 10.0%), online communities (Facebook: 6/40, 15.0% and Twitter: 5/40, 12.5%), and the Line app (4/40, 10.0%). Figure 3 presents the detailed recruitment process.

Table 1 presents comparisons among the MSM tested without referrals, index subjects, and tested sexual partners. As indicated, the mean age (*F*_2_=6.9, *P*=.001) and age of sexual debut (*F*_2_=5.5, *P*=.005) of the index subjects and tested sexual partners were significantly lower than those of the MSM tested without referrals.

**Table 1:** Comparisons of participants’ demographics, risk behaviors, HIV-testing experiences, and testing results (N=393).

Variable		MSM^a^ tested without referral^b^ (n=191)	Index subject^c^ (n=75)	Tested sexual partner^d^ (n=127)	LSD test^e^	*F* value (*df*)	*χ*^2^ (*df*)	*P* value
Age (years), mean (SD)		32.7 (8.5)	29.5 (6.3)	29.9 (7.5)	b > c, d	6.9 (2)	N/A^f^	.001
**Education, n (%)**					N/A	N/A	4.6 (2)	.33
	Above university	37 (19.4)	16 (21.3)	20 (15.7)				
	College or university	133 (69.6)	48 (64.0)	83 (65.4)				
	High school or lower	21 (11.0)	11 (14.7)	24 (18.9)				
**Employment, n (%)**					N/A	N/A	3.7 (2)	.44
	Employed	140 (73.3)	55 (73.3)	83 (65.4)				
	Unemployed	26 (13.6)	11 (14.7)	27 (21.3)				
	Student	25 (13.1)	9 (12.0)	17 (13.4)				
**Marital status, n (%)**					N/A	N/A	1.9 (2)	.75
	Single	179 (93.7)	73 (97.3)	121 (95.3)				
	Married	9 (4.7)	1 (1.3)	4 (3.1)				
	Divorced	3 (1.6)	1 (1.3)	2 (1.6)				
**Sexual orientation, n (%)**					N/A	N/A	0.2 (1)	.89
	Homosexual	166 (86.9)	65 (86.7)	108 (85.0)				
	Bisexual	25 (13.1)	10 (13.3)	19 (15.0)				
**Preferred position, n (%)**					N/A	N/A	8.9 (2)	.06
	Versatile	100 (52.4)	32 (42.7)	62 (48.8)				
	Top	51 (26.7)	31 (41.3)	31 (24.4)				
	Bottom	40 (20.9)	12 (16.0)	34 (26.8)				
**Finding sexual partners through social media^g,h^, n (%)**					N/A	N/A	0.7 (1)	.72
	Yes	131 (68.6)	48 (64.0)	83 (65.4)				
	No	60 (31.4)	27 (36.0)	44 (34.6)				
Age of sexual debut (years), mean (SD)		21.5 (5.2)	19.7 (3.7)	20.0 (4.6)	b > c, d	5.5 (2)	N/A	.005
Number of sexual partners^g^, mean (SD)		2.1 (1.9)	3.1 (5.5)	2.9 (2.5)	b < c, d	3.8 (2)	N/A	.02
Number of times engaging in anal intercourse^h^, mean (SD)		4.0 (3.2)	6.8 (7.0)	6.1 (6.1)	b < c, d	10.1 (2)	N/A	<.001
**Frequency of condom use during anal intercourse^h,i^, n (%)**					N/A	N/A	7.97 (3)	.24
	Used every time	69 (41.1)	25 (39.1)	46 (42.2)				
	Frequently used	55 (32.7)	17 (26.6)	25 (22.9)				
	Rarely used	40 (23.8)	17 (26.6)	29 (26.6)				
	Never used	4 (2.4)	5 (7.8)	9 (8.3)				
**Experiences with recreational drug use, n (%)**					N/A	N/A	1.1 (1)	.55
	No	168 (88.0)	63 (84.0)	107 (84.3)				
	Yes	23 (12.0)	12 (16.0)	20 (15.7)				
**Experiences of HIV testing, n (%)**					N/A	N/A	6.1 (1)	.047
	Yes	176 (92.1)	64 (85.3)	106 (83.5)				
	No	15 (7.9)	11 (14.7)	21 (16.5)				
**Interval of regular HIV testing (n=176), n (%)**					N/A	N/A	15.4 (3)	.02
	Three months	41 (23.3)	18 (28.6)	14 (13.1)				
	Half year	62 (35.2)	21 (33.3)	41 (38.3)				
	One year	35 (19.9)	6 (9.5)	13 (12.1)				
	Not regularly tested	38 (21.6)	18 (28.6)	39 (36.4)				
**HIV-testing results, n (%)**					N/A	N/A	0.9 (1)	.64
	Negative	189 (99.0)	74 (98.7)	124 (97.6)				
	Newly positive	2 (1.0)	1 (1.3)	3 (2.4)				

^a^MSM: men who have sex with men.

^b^Those who did not refer or disclose any sexual partner.

^c^Those who agreed to refer or disclose their sexual partners during the first wave.

^d^Those who were referred through the Line app and completed the rapid HIV test by the trained staff.

^e^Fisher least significant difference test.

^f^N/A: not applicable.

^g^Including mobile instant messaging or geosocial apps and online communities.

^h^In the past 3 months.

^i^N=341; due to missing data.

Further, the mean number of sexual partners (*F*_2_=3.8, *P*=.02) and mean frequency of anal intercourse (*F*_2_=10.1, *P*<.001) during the preceding 3 months were significantly higher for both the index subjects and tested sexual partners than for the MSM tested without referrals. The percentages of those who had not previously undergone HIV testing (*χ*^2^_1_=6.1, *P*=.047) and did not regularly undergo testing (*χ*^2^_2_=15.4, *P*=.02) were also significantly higher among the index subjects and tested sexual partners than the MSM tested without referrals. The HIV-seropositivity rate of the tested sexual partners was 2.4%, which was higher than the rates for both the index subjects (1.3%) and the MSM tested without referrals (1.0%); these did not show statistically significant differences.

**Figure 4:**
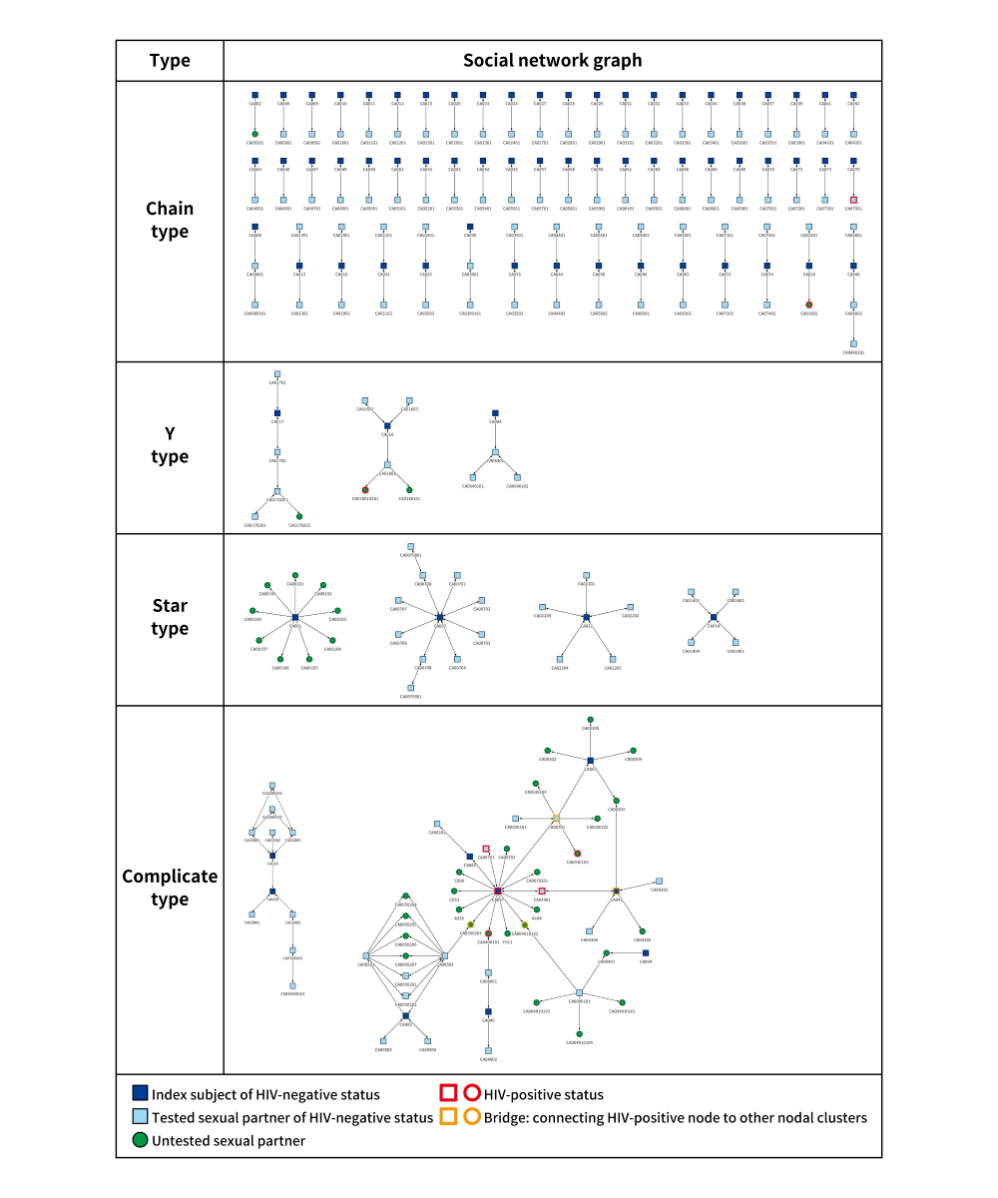
The social network graph.

Figure 4 shows the graph after partner-elicited interviews of 75 index subjects, to which were added 127 tested and 40 untested sexual partners. Three newly diagnosed HIV nodes and 4 previously known HIV-positive nodes were discovered, and this resulted in an HIV-positive rate of 4.2% (7/167) for all sexual partners. The sociometric structure in this social network graph included 59 pairs and groups of chain types (n=134). There were 3 Y-type groups (n=16), 4 star-type groups (n=32), and 2 groups of the complicated type (n=60). In the largest complicated type (n=48), the number of HIV-positive nodes was also the largest (n=5), and 4 sexual partners created a bridge with direct sexual affiliations between an HIV-positive node and the other nodal clusters, thus playing an important role as HIV transmission intermediaries. Of these, 2 tested bridges had been referred via the Line app and 2 untested bridges had been disclosed, namely the Hornet and Grindr apps, where the index subject first met the sexual partners.

Table 2 presents the network graph analysis of the number of intermediate nodes, which link 160 HIV-negative tested (n=124) and untested (n=36) sexual partners to the closest HIV-positive nodes (n=8). Of them, 73.1% (117/160) had no sexual affiliation with the HIV-positive node and 26.9% (43/160) had sexual affiliations with the HIV-positive node directly or through 1 to 3 intermediate nodes. Untested sexual partners reflected a higher proportion (10/25, 40%) of direct sexual affiliations with HIV-positive nodes than tested sexual partners (3/18, 17%).

**Table 2:** Data on intermediate nodes linking HIV-negative nodes of sexual partners to the closest HIV-positive nodes.

Variable^a^			Total HIV-negative sexual partners (n=160), n (%)		Tested sexual partners^b^ (n=124), n (%)		Untested sexual partners^c^ (n=36), n (%)
Not linking to any HIV-positive node			117 (73.1)		106 (90.6)		11 (9.4)
**Linking to the closest HIV-positive node**			43 (26.9)		18 (41.9)		25 (58.1)
	Directly	13 (30.2)		3 (16.7)		10 (40.0)	
	Through 1 intermediate node	11 (25.6)		7 (38.9)		4 (16.0)	
	Through 2 intermediate nodes	16 (37.2)		5 (27.8)		11 (44.0)	
	Through 3 intermediate nodes	3 (7.0)		3 (16.7)		0 (0)	

^a^Each node was calculated at the closest distance to an HIV-positive node; the calculation was not repeated.

^b^Those who were referred through the Line app and completed the rapid HIV test by our trained staff.

^c^Those whose accounts and profiles on social media platforms where they first met the referrer were disclosed by the referral to our staff.

## Discussion

### Principal Findings

This study’s main finding was that the partner-elicited interview successfully persuaded MSM to refer their sexual partners through the Line app to promote HIV testing. Eliciting referral via tested MSM could target sexual partners with behaviors carrying higher HIV risk. The data collected through this referral mode may contribute to understanding hitherto unknown social network affiliations.

The concept of helping and disclosing proved successful for developing and guiding the partner-elicited interviews in this research. Feedback from the referrers who had agreed to refer or disclose after the partner-elicited interviews indicates that they felt altruistic, had noncritical attitudes, and felt empowered to share the messages from the trained staff during the interview process. Moreover, the application of easy referral steps via the familiar and private interface [31] of the Line app was a key factor that motivated the referrers to be willing to refer their sexual partners for HIV testing. The interviews were conducted prior to HIV testing and subsequent test results. Referrers did not have to bear the negative consequences of notifying their sexual partners of an HIV diagnosis [32], which may have been a contributing factor to securing referrers’ cooperation.

All the sexual partners (127/127, 100.0%) referred via the Line app completed the rapid HIV tests by our trained staff during the research period, which signifies that the referral strategy was highly feasible and that the recruitment exercise had been successful. One possible reason for the high testing rate of the sexual partners could be the thorough preparation of the recruiters and the trust relationship between the referrers and peers [33]. We provided a suggested referral message to share with sexual partners to explain the reason for the referral and endorse the testing experience. The relationship tends to be more intimate than a causal relationship when the Line app is used to deliver private messages related to sexual behavior and HIV testing [34]. In addition, the homepage of our official Line account had a public and credible introduction [35], and both these methods increased the sexual partners’ trust to add the recommended Line account. Our model emphasized the web discussion of the HIV testing appointment individually in the private chat room of the Line app and a free, anonymous, rapid HIV test at a designated time and place, which contributed to the willingness of MSM to undergo the HIV test [17,36].

The partner-elicited interviews could be better used to target at-risk MSM and promote the HIV case findings. These results are similar to those of previous studies that used contact tracing and partner notification of HIV-positive index subjects in China [37,38]. Although the new HIV-positive rate of 10.5% to 11.1% among sexual partners in the previously mentioned study is higher than the rate of 2.4% in our study, our results still revealed that HIV case findings can be mobilized earlier among MSM via social networking platforms prior to an HIV-positive diagnosis. During the study period, Taiwan began to promote pre-exposure prophylaxis for the HIV risk group, accompanied by a large number of mobile screening programs in the community, which resulted in a sharp drop in the HIV-positive rate [39,40]. This may also be one of the reasons for the low positive rate in this study.

The accounts and profiles from social networking platforms used to refer/disclose sexual partners can be captured and exported to the network graph to present unknown and complex sexual affiliations [41]. This study uncovered a diverse network structure. The application of social network analysis (eg, multilevel modeling) is needed in the future to obtain insights into the different network structures. A high percentage of the tested MSM did not refer/disclose their sexual partners in this study, and we therefore cannot exclude the possibility that most HIV risk-taking information within the social network remains unknown. Moreover, it is worth noting that more untested sexual partners were at high risk of HIV infection via direct sexual affiliations with HIV-positive nodes. A survey also found that a high percentage (82.3%) of people newly diagnosed with HIV/AIDS had sex without a condom with an average of 2 sexual partners [42]. In the current network, 2 untested bridges need urgent testing and education on safe sex, thus helping to break the current HIV transmission risks affecting the nodal cluster [43]. However, due to the law of personal data protection in Taiwan, the trained staff are not allowed to contact the 2 untested bridges. Hence, the index subject forms an important channel through which health care providers may be able to provide an HIV self-test tool or link these sexual partners, who cannot be contacted otherwise, to testing resources. In addition to sexual partners’ referral, providing education to change risky behaviors, such as condomless anal sex, during pretest and posttest counseling may improve treatment and adherence to highly active antiretroviral therapy in the future [44].

Most participants in this study met new sexual partners through social media, which reflects the findings of previous studies [45]. In addition to continuously eliciting network information from MSM during interviews, it is important to develop innovative measures for health care providers to directly reach out and provide testing resources through these same applications and communities.

### Limitations

This study has several limitations. First, we directly recruited MSM who were willing to refer or disclose their sexual partners, which was not a random selection. This excluded many individuals who were unwilling to participate, thus affecting the study’s generalizability. Second, this study was conducted at a community screening station in Taipei City, which limits the inferences that can be made. Third, the social network was analyzed at the end of the data collection process, which resulted in static presentation. For this reason, causal relationships could not be confirmed. Fourth, the research period lasted only 6 months, which may have prevented a thorough exploration of the social networks. Future related studies should thus be conducted at multiple screening stations over a longer research period, thereby increasing the amount of data available to complete and analyze the social networks. Finally, there are currently no specific criteria to determine the validity of referrals. A comparison with traditional approaches is needed.

### Conclusion

Partner-elicited interviews could promote the referral/disclosure of sexual partners via social networking platforms among MSM. The motivational interviewing and referral process via the Line app used in this research can be directly applied in the education and practical training of HIV-screening consultants. Further, this may enhance the uptake of HIV testing and help target HIV-at-risk sexual networks. This study’s social network analysis revealed the types and unknown details of the direct and indirect sexual affiliations of each HIV-negative and HIV-positive node, thus promoting a better understanding of the possible distance of HIV transmission within the network. Emerging screening methods (eg, tests delivered through peer referrals or the direct recruitment of MSM through social media) are needed to actively target untested MSM engaging in condomless anal sex. These results may be useful for those attempting to improve current HIV screening programs and contact tracing for newly diagnosed case findings and surveillance.

## Appendix

Multimedia Appendix 1 The contents of partner-elicited interviews.

## Abbreviations

MSM: men who have sex with men
VCT: voluntary counseling and testing
