# Supplementary material for: Sexual Partner Referral for HIV Testing Through Social Networking Platforms: Cross-sectional Study
Source: JMIR Public Health Surveill. 2022 Apr 5;8(4):e32156. doi: 10.2196/32156 (PMC9019628; doi:10.2196/32156)
Supplement: Multimedia Appendix 1 [file publichealth_v8i4e32156_app1.docx]

**Multimedia Appendix 1.** The contents of partner-elicited interviews.

| The concept of helpfulness and disclosure | Empathy and inspiring statements of the referral | Guiding question |
| --- | --- | --- |
| Verbal persuasion | 1. We know that discussing and sharing the issues of sexual partners may cause uncomfortable feelings. We can discuss them according to your willingness to expose or not to expose. 2. Since you can complete the test later, referring your sexual partner to us for an HIV test could promote self-awareness about HIV status in your relationship. 3. An HIV test for your sexual partner could facilitate and create an opportunity to discuss safe sexual behavior or HIV preventive measures for both of you. | 1. What is your opinion about the use of persuasion when referring your sexual partner? 2. Would you consider to refer of your sexual partners for an HIV test? |
| Emphasized the benefits | 1. We understand that helping your sexual partner to get the HIV screening resources may not be easy to perform. We can consider the following advantages. 2. Two easy steps for you and your sexual partner to finish the refer and testing appointment via Line app. First, a suggested referral message will be provided to you to repost to sexual partners directly. Second, you can repost our QR code or share our account on Line app to your sexual partner. In sexual partners side, he can first preview our home page and decided to add our account as a friend or not. Then, the HIV testing appointment could be made and discussed on the chat room with our train staff. 3. Trained staff will provide a free, anonymous rapid HIV test to your sexual partner at a designated testing time and place for his convenient. | 1. In your opinion, will these benefits contribute to stronger wellness regarding involvement in this referral action? 2. Could you refer of your sexual partners for as using the way we suggested? |
| Motivated to be a helper | 1. We can feel that you must have thoughtful considerations if you are unwilling to refer of your sexual partner for the HIV testing. 2. It is beneficial to disclose information about your sexual partner to conducting a sexual network analysis, thereby helping to discover unknown HIV related sexual affiliation in the current MSM community to develop effective HIV prevention measures. 3. The information about your sexual partner, for example, the user account and profile on specific social networking platform, and whether you know his current HIV status can help us conduct an important social network analysis. | 1. What are your views on helping us and the MSM community through the mentioned method? 2. Could you help us by sharing the information of your sexual partners for revealing the social network and developing the HIV preventive measure? |

HIV= Human immunodeficiency virus; QR= Quick Response; app= application; MSM= men who have sex with men.
